# Miller Fisher Syndrome Associated With COVID-19: A History of Molecular Mimicry and an Up-to-Date Review of the Literature

**DOI:** 10.7759/cureus.43111

**Published:** 2023-08-08

**Authors:** Turan Poyraz

**Affiliations:** 1 Department of Elderly Care, Izmir University of Economics, İzmir, TUR

**Keywords:** miller fisher syndrome, sars-cov2, molecular mimicry, covid-19, anti-gq1b

## Abstract

Miller Fisher syndrome (MFS) was first recognized by Collier in 1932 as a clinical triad of ataxia, areflexia, and ophthalmoplegia. In 1956, three cases with this triad were published by Miller Fisher as a limited variant of Guillian-Barré syndrome (GBS), and the disease started to be called by his name. Since the beginning of the SARS-CoV-2 pandemic, there have been many reports of peripheral and central nervous system involvement. Until December 2022, a total of 24 cases, including four children associated with MFS, had been reported. This current review aimed to present the basic clinical and laboratory characteristics of patients with MFS and coronavirus disease-2019 (COVID-19). Since 2020, cases with different age and gender characteristics have been reported from eight different countries. Most cases were reported from Europe. SARS-CoV-2 infection was confirmed in seven of the cases. The youngest case reported was a 6-year-old boy from Turkey, while the oldest case was a 70-year-old female from Spain. All these reported cases and our past medical knowledge of MFS suggest that molecular mimicry is the main immunological mechanism. Despite all these data, more case reports, cohorts, and case-control studies will be needed to clarify the relationship between MFS and COVID-19.

## Introduction and background

Miller Fisher syndrome (MFS) was first described by Collier in 1932 as a variant of Guillain-Barré syndrome (GBS) characterized by ataxia, ophthalmoplegia, and areflexia [1]. In 1956, three cases with this triad were published by Miller Fisher, and the disease started to be called by his name [2]. The incidence of GBS is one to two per 100,000, and the MFS variant represents a small subset of these patients (5% of GBS) [3]. One of the key biomarkers of MFS patients is the presence of a normal white blood cell (WBC) count with typically elevated protein levels in the cerebrospinal fluid (CSF), which is termed albuminocytological dissociation (ACD). Another diagnostic marker of MFS is an anti-GQ1b IgG antibody against ganglioside GQ1b. In addition to these diagnostic markers, some findings can be observed in electroneuromyography (EMG) and magnetic resonance imaging (MRI).

Severe acute respiratory syndrome-coronavirus 2 (SARS-CoV-2)-associated coronavirus disease-2019 (COVID-19) was first documented in December 2019 in Wuhan, China [4]. COVID-19 was declared a pandemic by the World Health Organization (WHO) on March 11, 2020. Since the outbreak of the COVID-19 pandemic, many neurological symptoms, syndromes, and complications have been reported [5]. The most common neurological complaints in patients with COVID-19 are anosmia, ageusia, and headache. However, more serious adverse events such as stroke, impaired consciousness, seizures, and encephalopathy have also been reported [5].

By January 2023, a total of 25 cases of COVID-19-associated MFS have been reported, including four children [6-9]. The main neuroimmunological mechanism in MFS caused by both SARS-CoV-2 and other microbial agents is theoretically thought to be molecular mimicry or a maladaptive response to infection. As of December 23, 2022, more than 650 million patients had been diagnosed with COVID-19, causing more than six million deaths worldwide. Given the recent reports of cases of postvaccine MFS associated with COVID-19 vaccines and the presence of approximately 2.5 billion (13 billion doses) vaccinated individuals [10], it was aimed to systematically review all reported cases regarding the possible link between COVID-19 and MFS [11]. This review is also aimed to describe the temporal relationship of data on clinical, electrophysiological, and basic pathogenic mechanisms.

## Review

Method

Search Method

From the first COVID-19 report to December 31, 2022, COVID-19-related MFS cases were made using specific keywords on PubMed. These are "Miller Fisher syndrome" AND "COVID-19" OR "SARS-CoV-2". English full texts of all detected cases were accessed (only one notification was in Spanish, but we translated it to English as well). From these texts, data were entered into a template in which demographic data, clinical features, and laboratory characteristics could be determined. According to the criteria we used in this research, we found 64 studies from PubMed. We also found seven studies by checking the reference lists.

Studies with incomplete clinical data, reviews, and articles outside the scope of our study were excluded from the study, and 28 full-text literature was reviewed in line with our study purpose. Three of these were not included in the study analysis because the diagnosis of MFS could not be confirmed and the other one had an overlapping report. A total of 24 cases of COVID-19-associated MFS were included in the study.

Continuous variables, such as age and time between infection and time of onset of symptoms, were expressed as medians. For the diagnosis of MFS, clinical features, EMG findings, CSF findings, and which ones were used were analyzed.

Inclusion Criteria

The inclusion criteria for published studies included the following:

1) Diagnosis of COVID-19 (via PCR nasopharyngeal swab or serum antibody testing).

2) MFS was confirmed by clinical examination and diagnostic testing, including electrophysiological studies and cerebrospinal fluid (CSF) analysis.

The certainty of MFS diagnosis was assessed, based on the reported findings, by the Brighton Collaboration GBS Working Group criteria.

A level 1 diagnosis based on the Brighton criteria indicates the highest degree of diagnostic certainty supported by electrophysiological studies and the presence of albuminocytological dissociation (ACD) in the CSF.

A level 2 diagnosis was supported by electrophysiological data consistent with the polyneuropathy patterns described for GBS and MFS, either with a CSF white cell count of less than 50 cells/μl (with or without high protein level) or if CSF was not obtained.

A level 3 diagnosis is based on electrophysiological findings or clinical features not supported by CSF analysis.

Exclusion Criteria

The exclusion criteria for studies included the following:

1) COVID-19 patients diagnosed outside of MFS, such as myopathy, toxic-induced polyneuropathy, critical illness polyneuropathy (CIP), and critical illness myopathy.

2) Duplicate studies involving repetition of cases.

3) Studies in languages other than English (except those with abstracts in English).

**Figure 1:**
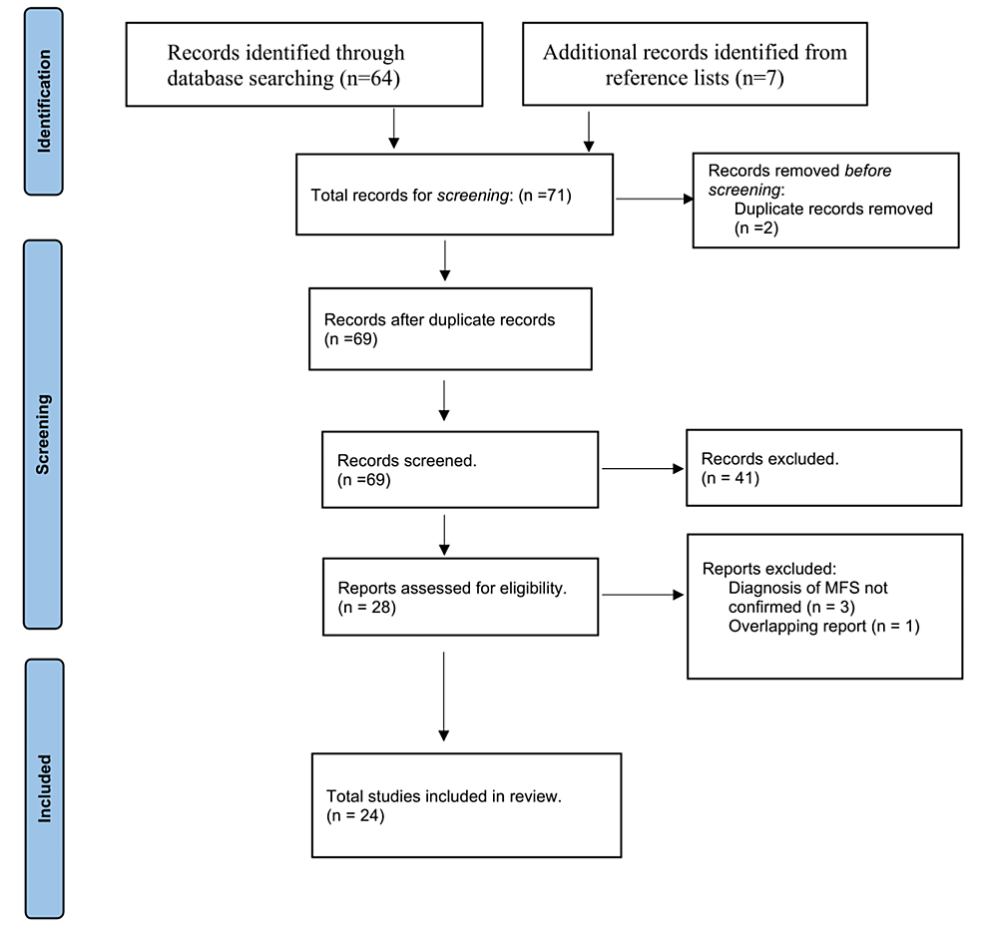
Flow diagram of studies included in the systematic review

This systematic review was conducted by using the Preferred Reporting Items for Systematic Reviews and Meta-Analyses (PRISMA) Statement (Figure 1).

Data Collection

From selected studies, we extracted the following variables for our analysis: age, gender, study type, country of origin of the case, the clinical presentation of MFS, RT-PCR for SARS-CoV-2 infection including nasopharyngeal and serum antibodies diagnostic tests, the delay between COVID-19 symptom onset and first symptoms of MFS, the presence of respiratory signs, EMG/NCS findings, the presence of ganglioside antibodies such as Anti-GQ1b, the presence of ACD in CSF, and the use of IVIG/plasmapheresis in treatment.

Data Analysis

Clinical characteristics were obtained as the number of patients, with the variable as the numerator and the total number of cases reported as the denominator: n/N (%). Continuous variables such as age were expressed as medians. The chi-square test was used for categorical covariates, and the t-test was used for continuous variables. The study data were analyzed using IBM Statistical Package for the Social Sciences (SPSS) (Windows 16.0) software.

Results

**Figure 2:**
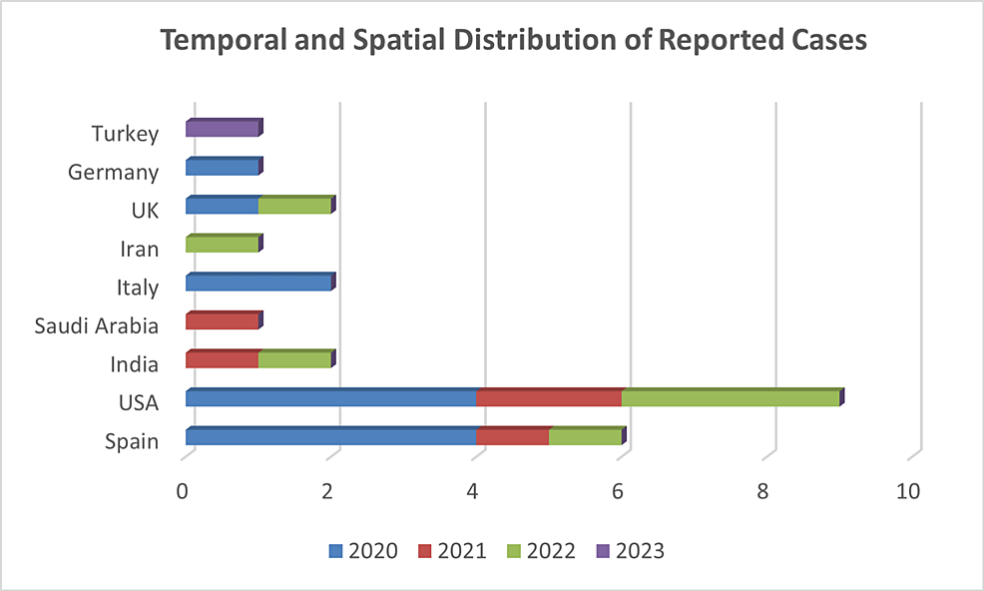
Temporal and spatial distribution of reported cases of COVID-19 associated with Miller-Fisher Syndrome in the literature from the first COVID-19 report to January 2023 The x-axis shows the number of described patients. The y-axis illustrates the countries of provenience of the cases. In each line, different colors represent the years 2020, 2021, 2022, and 2023 (until 7th January), in which the cases were published. Abbreviations: UK: United Kingdom, USA: United States of America

A total of 24 case studies were identified reporting 25 individual patients with MFS associated with COVID-19. The clinical, imaging, and laboratory findings of these 25 cases are summarized in (Table 1). The majority of the cases (n=19, 76%) were men and the median age was 40.32±18.87 (±SD) years. The first case, a male from Madrid, Spain, was posted online on April 17, 2020. The last reported case was a boy from Izmir, Turkey, reported in January 2023. Overall, the cases were from 9 countries. Most of the patients were from Europe (n=12, 48%), but most reports were from the USA (n=9, 36%). The temporal and spatial distribution of COVID-19-related Miller-Fisher Syndrome cases reported in the literature from the first COVID-19 report to January 2023 is shown in (Figure 2). All of the cases were diagnosed with COVID-19. Of these, only three patients had a positive antibody level against SARS-CoV-2 (tested during the absence of COVID-19 vaccines yet), and all the others were found to be positive for SARS-CoV-2 by quantitative RT-PCR in the nasopharyngeal swab. The diagnosis of COVID-19 was made before the onset of MFS in 23 cases (92%) and during hospitalization for MFS in the remaining two cases (8%). The most common symptoms were fever (n=18, 72%) and respiratory complaints (n=16, 64%). Other COVID-19 symptoms were taste and smell disorders and GIS findings, respectively. It was observed that MFS-related neurological findings started 14.56±15.58 days on average after the diagnosis of COVID-19 or the onset of symptoms. Among the classical triad findings, areflexia (n=24, 96%) was the most common. This finding was followed by ophthalmoparesis (n=22, 88%) and ataxia (n=22, 88%). It was observed that all 14 patients (56%) who underwent EMG had different pathological findings (such as delay of F wave latency, absence of H reflex, and conduction disorders). CSF analysis was performed in a total of 21 cases (84%), and ACD was found in 17 cases (77.27%). The test was positive in four (23.53%) of 17 cases in which anti-ganglioside antibodies were tested. Anti-GD1b was positive in only one of these cases, three other patients were anti-GQ1b positive, and the rest were negative. As immunotherapy, it was determined that 23 cases (92%) received IVIG treatment, and plasmapheresis was also applied to two cases (8.7%) who received IVIG. Spontaneous remission was observed in two cases (8%).

**Table 1:** Clinical, laboratory, and general findings of MFS patients GIS: Gastrointestinal system, UK: United Kingdom, USA: United States of America

Clinical, laboratory, and general findings of MFS patients	Number (%)
Gender	
Female	6 (24.0)
Male	19 (76.0)
Countries	
Spain	6 (24.0)
USA	9 (36.0)
India	2 (8.0)
Saudi Arabia	1 (4.0)
Italy	2 (8.0)
Iran	1 (4.0)
UK	2 (8.0)
Germany	1 (4.0)
Turkey	1 (4.0)
Neurologic findings	
Ataxia	22 (88.0)
Areflexia	24 (96.0)
Ophthalmoplegia	22 (88.0)
Autonomic dysfunction	5 (20.0)
Hypersomnolence	0
Bulbar weakness	0
COVID-19 symptoms	
Respiratory findings (cough, desaturation, dyspnea, etc.)	16 (64.0)
Fever	18 (72.0)
GIS findings	5 (20.0)
Taste or smell dysfunction	11 (44.0)
First clinical presentation	
COVID-19	23 (92.0)
MFS	2 (8.0)
Diagnosis of SARS-CoV-2 infection	
Nasopharengeal swap positive	22 (88.0)
Serological positive	3 (12.0)
Anti-ganglioside antibodies (Anti-gQ1b)	
Positive	3 (17.65)
Negative	14 (82.35)
Albuminocytological dissociation (ACD)	
Available	17 (77.27)
None	5 (22.73)
Immunotherapy	
Intravenous Immunoglobulin	23 (92.0)
Plasmapheresis	2 (8.33)
EMG findings	
Available	14 (56.0)
None	11 (44.0)

Discussion

MFS is an acute polyneuropathy characterized by the triad of ataxia, areflexia, and ophthalmoplegia. The worldwide incidence of GBS is approximately one to two in 100,000, with the MFS variant representing a tiny subset of the cases (one to two in 1,000,000) [3].

While some of the COVID-19-related MFS cases reported so far have severe COVID-19 infection to the point of hospitalization, many cases have been reported to have a mild clinical course of infection. Again, the fact that patients who test positive for COVID-19 have consistent presentations with MFS despite never having symptomatic COVID-19 highlights that the syndrome can potentially occur in any patient exposed to SARS-CoV-2, regardless of disease severity [11]. In this systematic review, we evaluated the clinical features of patients developing COVID-19 based on these case reports. However, larger epidemiological and large cohort studies are needed to understand the causal link between COVID-19 and MFS. Considering that COVID-19 is still spreading rapidly and remains contagious with different variants, more research will be needed to explore how COVID-19 may affect the nervous system. Further similar reviews and molecular-based studies will also enable us to understand the mechanism underlying the relationship between COVID-19 and MFS.

Antecedent infections, such as upper respiratory tract infection or gastroenteritis, are responsible for the pathogenesis of molecular mimicry [12]. The most common pathogens are *Campylobacter jejuni* and *Hemophilus influenza* [13]. However, *Cytomegalovirus, Mycoplasma pneumoniae, Ebstein-Barr virus, Varicella zoster, Coxiella burnetti, Pasteurella multocida,* and *Aspergillus* are also found to be associated [14-18]. However, in many cases, no causative pathogen has been determined [19]. Since the emergence of the COVID-19 pandemic, cases of MFS associated with COVID-19 have also started to be reported. The first case, a male from Madrid, Spain, was reported on April 17, 2020 [20]. In 2020, a total of 12 cases were reported from different countries of the world [20-30]. The number of reported cases in 2021 was five [7,9,31,32]. Seven cases were reported in 2022 [33-39]. The last reported case was a boy from Izmir, Turkey, reported in January 2023 [6]. Post-vaccination cases have also been reported in addition to antecedent infections. These are seasonal influenza vaccines [40-42]. Recently, MFS cases related to vaccines developed against COVID-19 have also started to be reported [10]. The demographic data and anti-GQ1b antibody positivity are summarized in (Table 2).

**Table 2:** Study origin, demographics, and GQ1b Ab *Anti-GD1b:+, UK: United Kingdom, USA: United States of America, N/A: Non-available, M: Male, F: Female

Study [reference]	Country	Age (yr)	Gender	No. of patient	COVID-19 symptoms	Anti-GQ1b
Poyraz [6]	Turkey	6	M	1	None	Negative
Raghunathan et al. [7]	Indian	7	M	1	Available	Negative
Alhaboob [8]	S.Arabia	11	M	1	None	N/A
Puppo et al. [9]	Spain	11	F	1	Available	N/A
Gutiérrez-Ortiz et al. [20]	Spain	50, 39	M, M	2	Available	+*/Negative
Lantos et al. [21]	USA	36	M	1	Available	Negative
Reyesbueno et al. [22]	Spain	51	F	1	Available	Negative
Ray [23]	UK	63	M	1	Available	N/A
Senel et al. [24]	Germany	61	M	1	Available	Negative
Manganotti et al. [25]	Italy	50	F	1	Available	Negative
Fernández-Dominguez et al. [26]	Spain	74	F	1	Available	Negative
Dinkin et al. [27]	USA	36	M	1	Available	Negative
Assini et al. [28]	Italy	55	M	1	Available	Negative
Kopscik et al. [29]	USA	31	M	1	None	+
Rana et al. [30]	USA	54	M	1	Available	N/A
Tran et al. [31]	USA	42	M	1	Available	N/A
Kajani et al. [32]	USA	50	M	1	Available	Negative
Faulkner et al. [33]	UK	66	M	1	Available	Negative
Kara et al. [34]	USA	43	M	1	Available	N/A
Kuang et al. [35]	USA	26	M	1	None	N/A
Yaqoop et al. [36]	Indian	22	M	1	Available	N/A
Aldabain et al. [37]	USA	39	F	1	None	+
Biswas et al. [38]	Spain	55	M	1	None	+
Mohammadi et al. [39]	Iran	30	F	1	Available	N/A

MFS is primarily a clinical diagnosis based on the classical triad of ataxia, areflexia, and ophthalmoplegia. However, the classic triad may not always be an initial finding. Diplopia and ataxia were responsible for one-third and one-fourth of initial symptoms, respectively [43,44]. Ophthalmoplegia is a clinically evident table with variable severity in almost all patients, with the most commonly involved cranial nerve being the oculomotor nerve in a ratio of 56% [43]. While ophthalmoplegia is mostly symmetric and bilateral, may be rarely unilateral [45]. Diplopia complaints are observed in approximately 38% of ophthalmoplegic/ophthalmoparesis cases. Another classic finding of the triad is areflexia, which can usually be observed in all deep tendon reflexes (DTRs), may go with a clinic that can be observed later in the lower limbs and later in the upper limbs. In the literature, areflexia was observed in approximately 81% of cases [43]. In a large MFS series consisting of 466 cases by Ito et al. [13], the DTRs were found to be normal in about 12% of cases.

**Figure 3:**
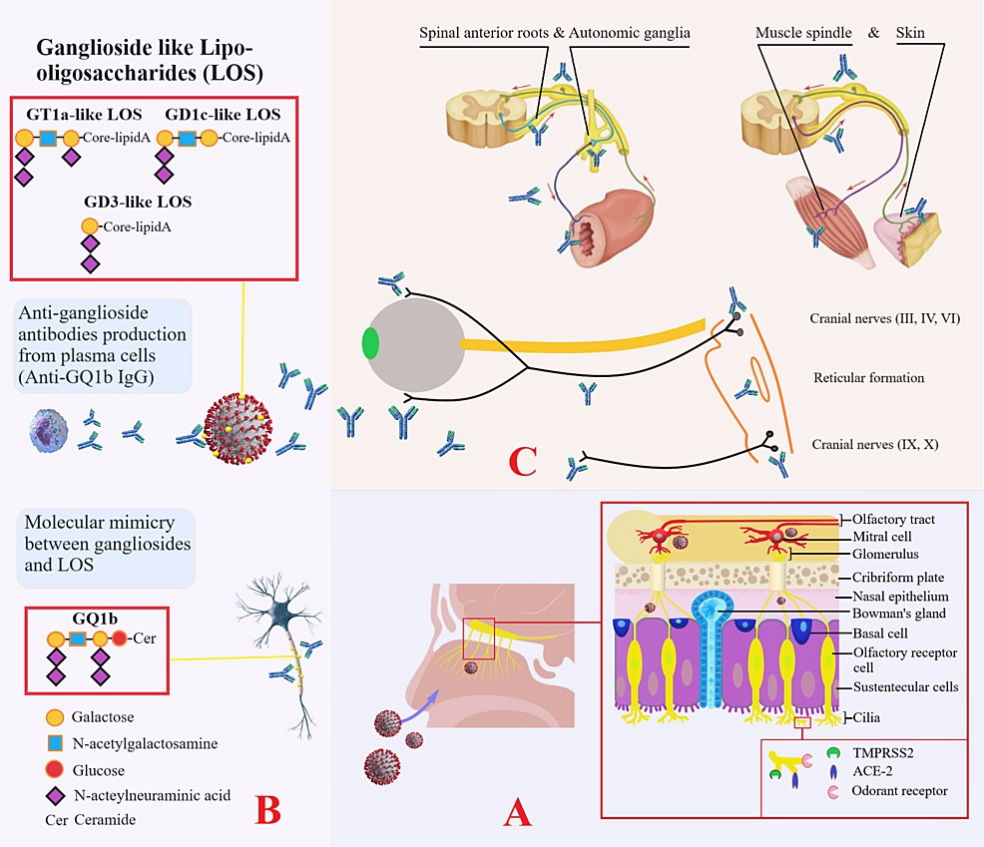
Mechanism of molecular mimicry in Miller Fisher syndrome associated with COVID-19 Panel A: Demonstrates transcribrial neurotropism of SARS-CoV-2 via ciliary chemoreceptors (TMPRSS2 and ACE-2 receptors) in the olfactory epithelium and neuroinvasion via olfactory tractus. Panel B: Demonstrates the mechanism of molecular mimicry between central and peripheral paranodal neuronal ganglioside epitopes and ganglioside-like lipo-oligosaccharides of the virus. Panel C: Demonstrates the antigenic targets of antibodies produced by plasma cells: muscle spindles; autonomic preganglionic, ganglionic, and postganglionic fibers; spinal anterior roots; third, fourth, and sixth cranial nerves; reticular formation; and ninth and 10th cranial nerves. The clinical features that occur as a result of the effects of these regions constitute Miller Fisher syndrome. This figure contains an original image created by the author.

MFS is a nodo-paranodopathy, and the main pathogenetic mechanism is thought to be molecular mimicry. Molecular mimicry between peripheral nerve and microbial/viral antigens is thought to occur through the activation of the adaptive immune system. Lewis rats with *Campylobacter jejuni* lipopolysaccharides were found to increase antibody formation against human ganglioside GD3 [46]. Similarly, the similarity of paranodal ganglioside epitopes with lipo-oligosaccharides on SARS-CoV-2 makes both the central and peripheral nervous systems a target for these antibodies. Although molecular mimicry is the dominant pathomechanism, recent studies have demonstrated the presence of the neuromuscular transmission defect associated with anti-GQ1b antibody formation both in vitro and in vivo [47]. Anti-GQ1b antibodies, which act against GQ1b (a ganglioside component of nerves), block acetylcholine release from motor nerve terminals [48]. It has been reported that anti-GQ1b antibodies may be positive and that CSF protein can be detected high in different series and case reports, especially in the early stages of the disease, but there are also serogroups with lower antibody positivity. Anti-GQ1b antibodies are detected as positive in approximately "85-90," especially in the MFS variant with ophthalmoplegia and GBS. Even though positivity can be seen in the MFS variant without ophthalmoplegia, it is not detected in GBS without ophthalmoplegia [49]. Here, the distribution of ganglioside epitopes in the paranodal regions of the fourth and sixth cranial nerves, mainly in the oculomotor nerves, is seen as the determinant. Considering this information, it is better understood why 85% of the positivity of anti-GQ1b antibodies can be seen in MFS cases in which external ophthalmoplegic involvement is the anterior plasma. Anti-GQ1b antibodies produced against the ganglioside epitopes present here cause conduction block by blocking impulse generation in the nodes of Ranvier [50]. The mechanism of molecular mimicry in MFS associated with COVID-19 is illustrated in Figure 3.

In the literature, atypical clinical features were observed in anti-GQ1b antibody-negative case reports. The majority of antibody-negative MFS cases were male, and the disease was observed at an earlier age [13,51]. Onset is rarely seen with diplopia, but it has been reported that, in the vast majority of antibody-negative cases in large case series, gastroenteritis precedes the clinical table [52]. It should be noted that antibodies such as anti-GT1a, GD1b, anti-GD2, and GD3 may be positive in anti-GQ1b antibody-negative cases [53-56].

A review of 123 patients with MFS found that 85% were positive for anti-GQ1b [57]. However, a systematic review in 2021 found that a majority of COVID-19-associated MFS cases had negative anti-GQ1b results [11]. There is an additional possibility that the positive anti-GQ1b results came from non-COVID-19 infections. The anti-GQ1b association and the exact pathophysiology of COVID-19 infection, leading to the demyelination of the peripheral nervous system, are still unclear and warrant further research. Interestingly, unlike MFS associated with COVID-19 infection, anti-GQ1b antibody positivity is detected more frequently in cases of MFS associated with COVID-19 vaccination (approximately 44%) [10]. While ganglioside autoantibodies are an important biomarker for non-COVID-19 MFS, they do not appear to be useful diagnostic biomarkers for patients with COVID-19-associated MFS [11]. As a hypothetical assessment of this situation, it is thought that arginylglycylaspartic acid (RGD) plays a role as an alternative receptor in cellular adhesion in the neuropathogenesis of COVID-19-associated MFS, and therefore different targets and immune-mediated mechanisms are involved [58].

ACD is characterized by elevated protein levels and normal cell counts in the CSF and may not be visible in the early stages of the disease. However, when it is detected, it is regarded as a meaningful finding in terms of disease. In COVID-19-associated MFS cases, ACD in CSF appears to be a more dominant finding rather than the presence of anti-GQ1b antibody. In addition, it has been determined that the MFS clinic can start weeks after the clinical symptoms of COVID-19, as well as simultaneously. This suggests that, during the incubation period of COVID-19, molecular mimicry and/or bystander activation cascade may be activated.

Electroneuromyography (ENMG) studies on MFS show various findings. The most consistent electrophysiological findings in MFS are reduced sensory nerve action potentials (SNAPs) amplitude and absent H reflexes. Sensory involvement is reported to be more frequent in nerve conduction studies (NCSs) [59]. NCSs of motor fibers are generally found to be normal, but findings of involvement (prolonged distal motor latencies (DML), the decreased amplitude of compound muscle action potentials (CMAPs), normal/slowing of motor conduction velocities (MCVs) are reported to be less frequent [59]. F wave latencies, known as late responses, and different results regarding H reflexes are reported, but the most common ENMG finding is the absence of H reflexes [10,20]. F-wave latencies have been reported to be normal, usually prolonged or absent. The absence of F waves has been interpreted as demyelinating conduction block in the proximal nerve segments when distal CMAPs are preserved [60]. In Durand et al.'s 10 MFS cases, ENMG studies showed that demyelinating and axonal changes were observed, and motor NCSs showed shrinkage and were mostly affected by F wave latency and H reflex loss [61]. The reporting of deterioration in nerve conduction studies, especially late responses (F wave and H reflex), in all 14 cases in which EMG examination was performed, showed that EMG, together with ACD, is an important diagnostic marker for COVID-19-related MFS.

Brain MRI was normal in almost all MFS cases; however, in some case reports, T2 hyperintense images were seen in the cerebral cranial nerves and posterior columns of the spinal cord, especially in the brain [62].

Both plasma exchange (PE) and IV immunoglobulin (IVIg) therapy have beneficial effects on GBS [63,64]. Immunomodulatory treatments were included in the treatment of MFS with the demonstration of significant curative effects after PE and IVIg [65,66]. However, patients with MFS usually do not require immunotherapy, presumably because they have a good prognosis and spontaneous recovery [67]. Theoretically, circulatory removal or neutralization of pathogenic antibodies should be beneficial in immune-mediated diseases. A retrospective clinical analysis conducted by Mori et al. [68], with 92 MFS patients suggested that IVIg treatment led to ophthalmoplegia and ataxia amelioration, but over time, these symptoms disappeared in similar to those of patients who received both PE and no immunotherapy. IVIg treatment is generally not necessary in MFS, except for BBSE or overlapping GBS, because it is costly and does not change the clinical outcome of patients.

Although MFS is usually a self-limiting disease, it may rarely show serious complications that lead to pulmonary arrhythmia and/or respiratory failure [69]. Ophthalmoplegia and ataxia begin to significantly improve in one to three months after the onset of the disease. However, a table of areflexia not associated with functional impairment may persist [70].

## Conclusions

MFS is a rare clinical entity that can be seen at any age and can be self-limited, spontaneous remission is common, and it can be seen more frequently in men at early ages. The clinical course occurring with ophthalmoplegia, and ataxia is expanding the spectrum of differential diagnosis. Multiple recurrence episodes can also be observed, usually uniform. It is important to differentiate between the disease BBSE and GBS, which can be diagnosed clinically, immunologically, radiologically, and neurophysiological. It should be known that the neurological complications of COVID-19 may start quite early or may occur after a long time, and therefore it should be considered that it would be useful to follow up the clinical conditions with periodic controls. It should be kept in mind that clinical manifestations, such as GBS and MFS, may also develop due to both COVID-19 itself and vaccines developed against COVID-19. Anti-GQ1b antibodies are important, but serology can be negative in the majority of COVID-19-related cases of MFS. The presence of ACD in CSF with EMG examination seems to have a higher diagnostic value for COVID-19-associated MFS.
